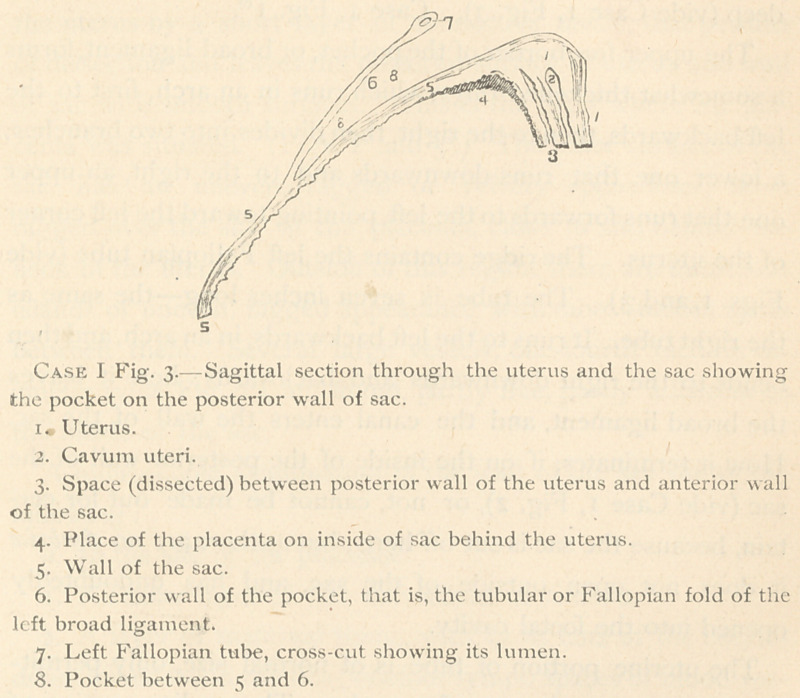# Report of the Gynecological Society of Chicago on Two Cases of Extra-uterine Pregnancy from Examination of the Specimens

**Published:** 1885-03

**Authors:** Christian Fenger

**Affiliations:** Surgeon to Cook County Hospital, Professor of General Pathology and Pathological Anatomy, Chicago Medical College. Member of the American Surgical Association


### ARTICLE II.

### REPORT TO THE GYNECOLOGICAL SOCIETY OF CHICAGO ON TWO CASES OF EXTRA-UTERINE PREGNANCY FROM EXAMINATION OF THE SPECIMENS *presented by* CHRISTIAN FENGER, M. D., *Surgeon to Cook County Hospital, Professor of General Pathology and Pathological Anatomy, Chicago Medical College. Member of the American Surgical Association.*

[Read before the Chicago Gynecological Society, Feb. 20th, 1885.]

*Mr. Chairman and Gentlemen of the Society:* The exact anatomical diagnosis or minute classification of an extra-uterine pregnancy is easy enough in the early stages of the disease, but it becomes more and more difficult in the latter half and towards the termination of the pregnancy.

In the earliest months of the pregnancy, it is only by accident that a pathological specimen is found. Here the exact diagnosis is easy enough. From the third to sixth months, specimens are secured by operation or after death, as the result of hæmorrhage from rupture, and here the diagnosis is still comparatively easy.

In the latter half of pregnancy, from sixth to tenth months, the diagnosis, viz., exact location of fecundated ovum, becomes often exceedingly difficult, next to impossible, on account of secondary changes, and often partial destruction of Fallopian tubes and ovaries, and still more difficult, if a fatal peritonitis has contributed to mask the normal anatomical features of the organs in question.

The two specimens, sent to me for examination, belong to the class of late and consequently difficult cases, and in one of them the specimen was very much decomposed. Nevertheless, I think that a close examination of the specimens permits of a comparatively exact classification of the two cases; at least, of one of them.

Before describing and demonstrating the specimens, permit me to recall to your memories the different forms of extra-uterine pregnancy.

*Extra-uterine Pregnancy.*—The ovum is arrested somewhere in its normal passage from the Graafian follicle down to the *cavum uteri*, or drops out of the passage, without or after rupture of the latter, into adjoining cavities or spaces.

1. *Ovarian Pregnancy.*—The ovum remains in the ovary. *Epi-ovarian Pregnancy.*—The ovum develops on the ovary, having left the Graafian follicle.

2. *Abdominal or Peritoneal Pregnancy.*—The ovum falls down into the peritoneal cavity, and does not enter the Fallopian tube at all.

3. *Tubal Pregnancy.*—

I. Tubo-abdominal or tubo-ovarian pregnancy.

II. Tubal pregnancy.

III. Tubo-uterine, or interstitial or mural pregnancy.

4. *Extra-peritoneal Pregnancy* in the broad ligament after rupture of the Fallopian tube.

5. Pregnancy in one side of a *uterus bicornis.*

6. *Secondary Abdominal or Peritoneal Pregnancy.*—Ovary, tube, or even uterus (*bicornis or normal*) is ruptured, the fœtus slips into the peritoneal cavity, but remains in connection with the primary sac.

I shall first describe and demonstrate Professor Byford's

CASE, NO. 2.

The uterus is large, four and a half inches long, three inches broad, at the fundus; the cavity also considerably enlarged. In *left* side of uterus and vagina, I find an incision opening, three and a half inches long, closed with silk sutures, leading from the uterus and vagina into the sac, or as some members of the Society called it, the adventitious uterus. The sac can only be seen in fragments. Its wall is one to two lines thick, the outside partly covered with peritoneum, partly adherent to the surrounding organs, viz., bladder, uterus, omentum. The rectum I do not find. Right ovary and Fallopian tube are missing.

The left Fallopian tube shows the following conditions: the uterine portion is of normal size, passable only for a thin probe, one-half mm. in diameter; at the distance of one-half inch from the fundus, it is wider, one-eighth of an inch in diameter, and so it continues for four inches; then it suddenly dilates to one inch in diameter, continues so for one inch, and thereafter opens into the fœtal sac, the wall of the latter going continuously over into the wall of the tube.

The left ovary cannot be found. Large shreds of the membranes of the ovum, viz., amnion and chorion, adhere to the sac here and there. Inside of sac is dark, brown-spotted, the color of decomposed blood. This condition is most pronounced in the part of the sac that covers the posterior wall of the bladder and the anterior and posterior walls of the uterus. On the uterus, the sac is thinner and more adherent (no sub-serous connective tissue) than on the bladder, where the wall of the sac is about two mm. in thickness, firm and movable against the bladder.

From the condition in which we find the left Fallopian tube, I think it safe to conclude that the ovum has developed in its outer half, near the abdominal end of the tube. The funnel-shaped dilatation of the tube in this place and the thickening of its wall, which continues uninterruptedly as the wall of the fœtal sac, prove the connection between the two cavities, and this case of extra-uterine pregnancy would thus be of the *tubo-abdominal* variety. I believe that the ovum has commenced its development in the tube, and then, with or without rupture of the latter, has formed its sac on the surface of the pelvic and surrounding abdominal organs. In this respect, it might be classified as a secondary abdominal or peritoneal pregnancy, originating in the abdominal end of the left tube.

PROFESSOR BYFORD'S CASE, NO. 1—This case has a greater interest, partly because the specimen is in a good state of preservation, and partly because some of its features seemingly point to one, others to another of the varieties of abdominal pregnancy.

In this case, as will be remembered, laparotomy was performed, and part of the cyst, the placenta and the upper two-thirds of the uterus removed. I shall not undertake, here, to describe the child, as it is irrelevant to the matter in question.

We find the uterine appendages of the right side, viz., broad ligament, round ligament, Fallopian tube and ovary normal, (vide Case 1, Fig. 1.)

CASE I Fig. 1.—Anterior view of uterus, its appendices and the extra-uterine sac.

1. Uterus laid open, fine silver wires passed through the uterine portion of both Fallopian tubes.

2. Right Fallopian tube.

3. Right round ligament.

4. Right ovary.

5. Left round ligament, enlarged but in normal position, viz., commencing at upper corner of uterus.

6. Ridge or the upper free border of the pocket (10) containing the left Fallopian tube through which silver wires (dotted lines) are passed. It is seen that the Fallopian tube only passes through the first two-thirds of the ridge, and then branches off, backwards and downwards (dotted line) to penetrate into the wall of the sac.

7. Terminal end of the ridge—probably the left ligamentum ovarii.

8. The sac.

9. Place of the placenta.

10. Pocket on upper posterior wall of sac.

the inner layer because the wall has been cut obliquely here at the operation.

11. Probe in the left Fallopian tube.

12. Oval opening on the inside of the outer layer of the sac, viz., termination of the Fallopian tube or cross-cut of the Fallopian tube just before it (probably) opened into the cavity of the extra-uterine sac.

The uterus, amputated about the middle of the neck, is of normal size, viz., the cavity, one and one-quarter inches between the two uterine orifices of the Fallopian tubes. Farther down, one inch, still lower down one-half inch, and in the neck one-fourth inch, broad. The average thickness of the uterine wall is one-half to three-fourths inch. To the left and behind the uterus and in uninterrupted connection with its surface is the sac or adventitious uterus. Fig. 1 shows the uterus and sac seen from the anterior side. From the anterior surface of the sac, one-fourth inch from the left corner of fundus, is the left round ligament; it is enlarged, one-fourth inch in diameter. On the upper surface of the sac, behind and to the left of the *fundus uteri*, is a pocket covered with peritoneum, two and one-half inches broad, three to three-and-a-half inches deep (vide Case 1, Fig. 3). Case 1, Fig. 1[10].

The upper free border of the pocket, or broad ligament, forms a somewhat thickened *ridge*, which runs in an arch, first to the left backwards, then to the right, then divides into two branches, a lower one that runs downwards and to the right, an upper one that runs forwards to the left, pointing toward the left corner of the uterus. The ridge contains the left Fallopian tube (vide Figs. 1, and 3). The tube is seven inches long—the same as the right tube. It runs to the left backwards, in an arch, and then bends to the right downwards and backwards; here it leaves the broad ligament, and the canal enters the wall of the sac. How it terminates, if on the inside of the posterior wall of the sac (vide Case 1, Fig. 2), or not, cannot be made out for certain, because the sac is cut off here; but as there are no *fimbriæ* it does not open outside of the sac, and has undoubtedly opened into the foetal cavity.

The uterine portion of tube is of normal size, only permitting the passage of a very fine probe. The median portion of the tube is normal, perhaps slightly dilated, three to five lines wide.

The termination of the tube in the wall of the sac, Fig. 2[12], is an oval opening, one-fourth inch in diameter, the borders of which are perfectly smooth, no *fimbriæ* visible anywhere.

Of the left ovary, no trace can be found. The sac is clad on the outside with the peritoneum, and is smooth.

The wall of the sac is from one to eight mm. thick, white and firm. The thickest part of the sac is right behind the *fundus uteri*, one-fourth to one-half inch in thickness, and there the tissue, viz., fibers of the uterine tissue of upper surface of fundus, is continuous with the wall of the sac; however, on the posterior surface of neck and fundus, the tissue of uterus is *not* continuous with the sac, but the latter is separated from the uterus by a short layer of connective tissue, that permits of dissection and leaves the posterior surface of uterus and wall of sac with smooth surfaces. This is the place where the placenta was situated. (Figs. 2[7] and 3[4].) The inner surface of the sac has an uneven, ragged or velvety appearance, most ragged over the site of the placenta, close to and behind the neck of the uterus. Outside of this region, there are numerous islands of uneven, ragged appearance, with more smooth spots between them. Several large vessels, one-fourth inch in diameter (vide Fig. 2[9]), are found, partly free, partly adherent to the inside of the sac.

A microscopic examination of the wall of the sac shows the following:

*a.* In the site of the placenta.

1. An inner layer of free cotyledons or *fimbriæ.*

2. A layer of maternal tissue, with cross cuts of the cotyledons, imbedded in cavities (whether lymph spaces or blood vessels, I am unable to decide, but they look to me like venous blood vessels) tightly surrounding them.

3. A heavy layer of connective tissue bundles, interspersed with some organic muscle bundles.

4. Peritoneum.

*b.* A thick spot in the wall, near the peripheral opening of the Fallopian tube into the sac, which I examined to find ovarain tissue, presents exactly the same appearance as *a.*

*c.* A thin place in the sac, some distance from placenta and tube, gives,

1. An inner layer of areolar connective tissue without cotyledons.

2. A median heavy layer of connective tissue bundles and bundles of organic muscle fibers.

3. Peritoneum.

*d.* A third place in the sac presents the same layers as *a.*

Nowhere in the wall of the sac, is any trace of ovarian tissue to be found.

In considering the anatomical diagnosis of this case, I shall have to take into consideration mural, ovarian, tubo-ovarian and tubo-abdominal pregnancy.

1. *Can it be a mural or interstitial pregnancy?* The continuity of the sac (in the site of the placenta) with the upper surface of the fundus belongs to the signs of mural pregnancy. The uterine portion of the Fallopian tube is of normal length and width, consequently, the ovum cannot have lodged and developed here. However, a persisting "Gärtner's duct" might, perhaps (it is doubtful), form a lateral branch of the tube and run in the wall of the uterus. Baudelocque, "the nephew," claims that a mural pregnancy can take place when the fecundated ovum lodges in this blind branch. Kleinwächter, in his article, "Tubal Pregnancy," in Eulenburg's Encyclopedia, remarks that this statement of Baudelocque's has yet to be proved.

Aside from Gärtner's duct, there is another anatomical anomaly that might give rise to a mural pregnancy, outside of the uterine portion of the Fallopian tube. Through the kindness of Professor Jaggard, of Chicago, my attention was called to this variety. The ovum may develop in a branch of a bifurcated Fallopian tube. Hennig* (Die Krankheiten der Eileiter und die Tubar-schwangerschaft) has an illustration showing a canal branching off from the Fallopian tube in the lateral wall of the uterus; the branch turns downwards and inwards in the uterine

* Lusk—"The Science and Art of Midwifery."

wall, and opens into the cavity of the uterus at the internal os. If the ovum is arrested in this branch, a mural pregnancy results. The sac will push the broad ligament and the appendices outwards and upwards, and we will expect, as in the ordinary mural pregnancy, to find Fallopian tube and ovary on the outside of the sac, and further, we must expect that the round ligament should be dislodged outwards some distance from the border of the uterus. Consequently, in our case, we cannot admit a bifurcated Fallopian tube as the seat of the pregnancy.

But supposing a mural pregnancy had taken place here, and consequently the uterine portion of the Fallopian tube could be found open outside of the sac : then we demand in this case certain conditions, that cannot very well be dispensed with, and they are the following :

The abdominal end of the Fallopian tube, together with the ovary, must be found somewhere on the outer wall of the sac, and opening into the peritoneal cavity.

Supposing that the ovary, for some reason or other, was not found and the abdominal end of the Fallopian tube was obliterated and buried in the wall of the sac, we might yet have had a mural pregnancy.

In this case, however, the Fallopian tube opens into the wall of the sac. If it has opened into the fœtal cavity, it can not be seen on this specimen. (However it looks as if it had probably done so.)

The round ligament in mural pregnancy is expected to be pushed outwards some distance from the side of the uterus. This might be different if the ovum could develop in the posterior wall of the uterus; but this possibility has never been proved. Gärtner's duct does not run in the posterior wall, but from the parovarium first in the broad ligament (in the same fold as the Fallopian tube), then in the muscular substance of the lateral border of the uterus and down on the side of the vagina where it terminates blindly.

The sac can be dissected off from the posterior wall of the neck and *fundus uteri*, which looks as if the sac developed on the posterior surface and not in the posterior wall of the uterus.

Thus, although the positive proof against mural pregnancy, viz., the opening of the Fallopian tube into cavity of the sac, is wanting (the fault of the specimen), then as all signs of mural pregnancy are absent, except the apparent continuity of sac wall and uterus, I shall declare against mural pregnancy.

The microscopic examination of Dr. Byford's Case (No. I), (our second Case) does not give any points as to the solution of the question of mural or tubo-ovarian pregnancy; the sac here consists just exactly of the same elements as I have found in a case of abscess of the broad ligament, in a wall as thick as the sac in its thickest parts. The presence of organic muscle-fiber in the sac, and the continuity or connection between the muscle-fiber of sac and uterine wall is of only secondary diagnostic significance, for the following reason: the organic muscle fiber or cell belongs to the *proletaires* (so to say) amongst the tissues; it is of the connective tissue class, can be formed and found everywhere where connective tissue is formed and found. In fibro-myomata or myo-fibromata, it is often impossible to determine what is a muscle cell and what is a spindle-shaped connective tissue cell. Consequently, continuity between muscle-fiber in sac and muscle-fiber of the uterus does not mean that the former originated in the latter.

The next question then is this: Is it an ovarian, tubo-ovarian or tubo-abdominal pregnancy?

In an ovarian pregnancy, we require, (1.) that the Fallopian tube does not participate in the formation of the sac (Kleinwächter). (2.) Ovarian tissue is found in the wall of the sac.

(3.) That there is a connection between the sac and the uterus through the *ligamentum ovarii.*

Of a tubo-ovarian pregnancy, we would require (1), that the peritoneal end of the Fallopian tube participates in the formation of, that is, opens into the sac, (2), the ovary may be intact, but it may also have been used up in the formation of the sac and have disappeared either entirely or only remnants may be found in the wall of the sac.

(It is easy to see how difficult it may be to find microscopic remnants of ovarian tissue in the wall of a sac one hundred times or more the size of a normal ovary).

As near as we, in my opinion, are able to to come to an exact diagnosis in this case, I should pronounce it a *tubo-ovarian pregnancy.*

The exact location of the spot where the fecundated ovum has commenced development, it is of course impossible to prove to satisfaction. Still there is one interesting feature in this case, which, in my opinion, throws some light on this point. This is the pocket, the blind pocket, on the upper wall of the sac behind the uterus, (vide, Fig. 1[10], and 3[8].) As before stated, the upper side of the posterior wall of the pocket, viz., the *ligamentum latum,* or the Fallopian fold of this ligament, forms a circular figure, commencing at the left border of the fundus and terminating at about the same point; from the connection between the middle and outer third, a branch goes off downwards and to the right. The Fallopian tube is contained in the first two-thirds of the ridge and in the branch. The final third of the ridge, that does not contain the tube, but runs back towards the left corner of the uterus, would, in my opinion, correspond with the *ligamentum ovarii.* (Case I, Fig. 1[7]) The formation of the pocket, clad with the peritoneum and having as upper border, the above-described ridge, can, in my opinion, be explained if the ovum has been arrested and commenced development in the *ligamentum* "*infundibulo-ovarianum*" (Henle) between the *fimbriæ* that line the sulcus leading from the distal end of the ovary to the *ostium abdominale* of the tube. If the ovum is developed here, it can, with the vessels of the chorion, first, reach the abdominal ostium of the tube, and thus permit the tube to open into the sac. Second, it can reach down on the lower or posterior surface of the ovary, and thus during its growth lift up the ovary at the same time as it destroys it, but in lifting it up, preserve and enlarge the peritoneal fold or pocket that normally exists between the posterior surface of the peritoneal fold containing the Fallopian tube and the anterior surface of the peritoneal fold containing the ovary and the *ligamentum ovarii.*

In case the fecundated ovum, from the ruptured Graafian follicle, had dropped down below the ovary, and had been arrested or taken hold on the peritoneal surface of Douglas' fossa or on posterior surface of ovary, if a development in such a way and place is possible, the pocket could be formed of course, but we could not expect to have the Fallopian tube open into the wall of the extra-uterine sac. If the pocket in question is formed in cases where the ovum has been arrested in the peripheral end of the tube, I do not know, as my access to original literature on this subject has been extremely limited, and the common text and hand-books, of course, do not contain anything like a detailed description of any of the cases in question.

In the proceedings of the meeting of the Gynecological Society of December 19th, 1884, Professor W. H. Byford is recorded to have said, (CHICAGO MEDICAL JOURNAL AND EXAMINER, Jan., 1885, p. 64) as follows: in the first Case (our Case No. II, where operation was performed), he thought that the fecundated ovum had passed through the tube, but found some diverticulum in the uterine cavity, had passed into the uterine wall and developed in this region, pushing the wall before it. The muscular element of the sac was directly continuous with the uterine muscle. Further, p. 64–65: "It is not necessary for the production of mural pregnancy that the tubes be involved."

*A Diverticulum* in the uterine wall that would permit the ovum to develop down between the muscle in the wall, is as far as I know, not known or proved, but such a condition might be accepted in a proved mural pregnancy in which the Fallopian tubes were not involved.

A Gärtner's Duct, as place of development for the ovum, is not proved either. But accepted as a possibility, let us see what would be the consequence. The ovum would be arrested either in the extra- or the intra-uterine portion of the duct. (I do not know of any communication opening between Gärtner's Duct and the Fallopian tube; does it exist?) If developed in the *extra-uterine portion* of the duct, that runs in the Fallopian fold of the broad ligament, the formation of the pocket in this Case (No. I) would be impossible. The ovary might disappear and the tube might run in the wall, but ought to open into the abdominal cavity.

If developed in the intra-uterine portion of Gärtner's Duct, we would expect to have the Fallopian tube and the ovary intact on outside of sac, just the same as in mural pregnancy from the uterine portion of the Fallopian tube. How great value in diagnosis of mural pregnancy, the fact has, that the muscle of the uterus is continuous with the muscle of the sac, I do not know; but in this case, apparently only the layers of the surface of the uterus are continuous with those of the sac, and in mural pregnancy I would rather expect to have the deeper layers participate also.

In conclusion, I shall proffer my thanks to the Society for the honorable task entrusted to me, and ask its pardon in that the material in question, and the literature at my disposal have not enabled me to give a more satisfactory report of the matter.

214 East Ohio Street, Chicago.

## Figures and Tables

**Case II Fig. 1. f1:**
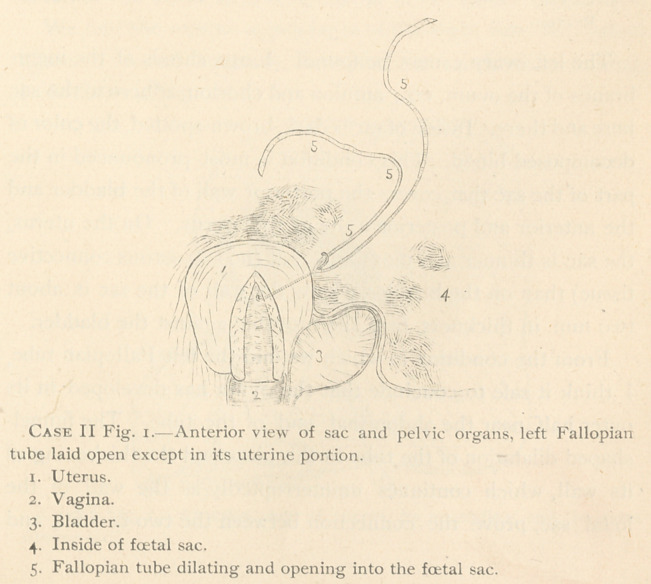


**Case II Fig. 2. f2:**
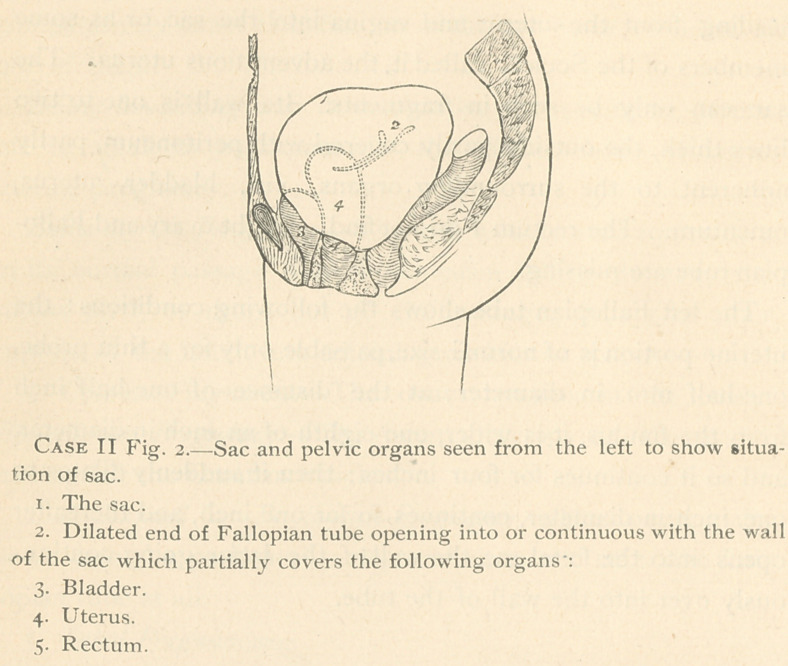


**Case I Fig. 1. f3:**
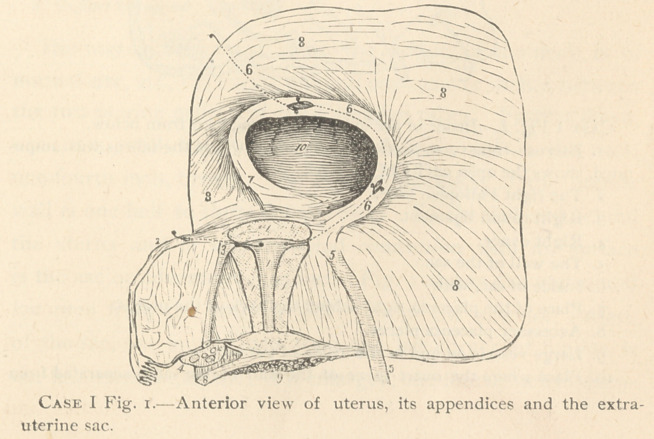


**Case I Fig. 2. f4:**
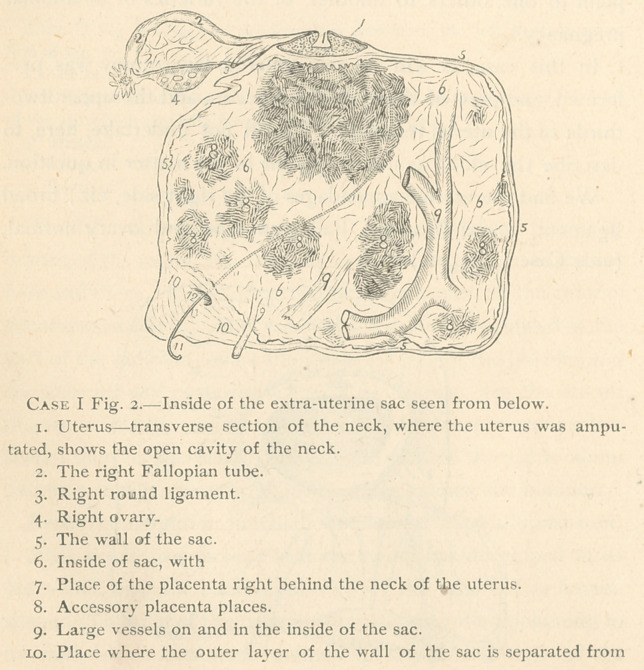


**Case I Fig. 3. f5:**